# Celecoxib and rofecoxib have different effects on small intestinal ischemia/reperfusion injury in rats

**DOI:** 10.3389/fphar.2024.1468579

**Published:** 2024-11-08

**Authors:** Szilvia B. László, Barbara Hutka, András S. Tóth, Tamás Hegyes, Zsuzsanna O. Demeter, Arezoo Haghighi, Gerda Wachtl, Ágnes Kelemen, Anna Jakab, Klára Gyires, Zoltán S. Zádori

**Affiliations:** ^1^ Department of Pharmacology and Pharmacotherapy, Semmelweis University, Budapest, Hungary; ^2^ Pharmacological and Drug Safety Research, Gedeon Richter Plc, Budapest, Hungary; ^3^ Department of Histopathology, Central Hospital of Northern Pest – Military Hospital, Budapest, Hungary; ^4^ Faculty of Information Technology and Bionics, Pázmány Péter Catholic University, Budapest, Hungary; ^5^ Center for Pharmacology and Drug Research and Development, Semmelweis University, Budapest, Hungary; ^6^ Department of Pathology and Experimental Cancer Research, Semmelweis University, Budapest, Hungary

**Keywords:** cyclooxygenase-2, celecoxib, rofecoxib, intestine, ischemia/reperfusion injury

## Abstract

**Introduction:**

Intestinal ischemia/reperfusion (I/R) injury is associated with high mortality and there is an unmet need for novel therapies. The intestinal expression of cyclooxygenase-2 (COX-2) increases rapidly after mesenteric I/R, but it is still a question of debate whether selective COX-2 inhibitors can mitigate I/R-induced gut injury. Here we aimed to compare the effect of celecoxib and rofecoxib, two selective COX-2 inhibitors, on intestinal I/R-induced injury in rats.

**Methods:**

Wistar rats were treated with celecoxib (10 and 100 mg/kg), rofecoxib (5 and 50 mg/kg), or vehicle for 8 days via gavage and then were subjected to sham operation or mesenteric I/R. Small intestinal inflammation and tissue damage were assessed by histology and quantification of inflammatory and tight junction proteins. The intestinal activity of COX enzymes was determined by a COX activity assay.

**Results:**

The higher dose of celecoxib reduced the I/R-associated increase in inflammatory mediators (myeloperoxidase, pentraxin 3, COX-2, interleukin-1β) and loss of tight junction proteins (claudin-1, occludin), whereas the lower dose of celecoxib was only marginally effective. However, even high-dose celecoxib failed to prevent the histological injury of the mucosa. In contrast to celecoxib, rofecoxib did not affect intestinal inflammation and injury at any of the tested doses. Neither celecoxib nor rofecoxib affected the I/R-induced changes of HO-1 and PPAR-γ, known off-targets of COX-inhibitors, but celecoxib increased the I/R-induced elevation of Bax/Bcl-2, a marker of apoptosis, whereas rofecoxib reduced the elevation of phospho-Akt. Importantly, high-dose celecoxib, but not rofecoxib, has already reduced intestinal COX-1 activity.

**Conclusion:**

Our study provides evidence for the higher anti-inflammatory efficacy of celecoxib compared to rofecoxib in mesenteric I/R injury, which is likely due to its lower selectivity for COX-2. However, even high-dose celecoxib was unable to reduce the mucosal damage. Our results suggest that selective COX-2 inhibitors have only limited therapeutic value in intestinal I/R injury.

## 1 Introduction

Intestinal ischemia can occur due to a variety of pathological conditions, such as mesenteric thrombosis, abdominal and thoracic vascular surgery, neonatal necrotizing enterocolitis, sepsis, or hypovolaemic shock (Mallick et al., 2004). As in any other organ, the lack of oxygen and nutrient supply induces multiple intracellular effects that ultimately lead to cell death. Because the severity of tissue damage depends on the magnitude and duration of ischemia, rapid restoration of blood flow is the mainstay of therapy to limit ischemic injury. However, reperfusion can paradoxically exacerbate tissue damage by generating reactive oxygen species, endothelial dysfunction, and inflammation (Carden and Granger, 2000; Kalogeris et al., 2012; Slegtenhorst et al., 2014). Moreover, by disrupting epithelial cells and their tight junctions, intestinal ischemia/reperfusion (I/R) can induce mucosal barrier dysfunction and translocation of luminal bacteria into the systemic circulation, potentially culminating in sepsis and multiple organ failure (Mallick et al., 2004; Bertoni et al., 2018).

Because of the complex pathogenesis of intestinal I/R injury and lack of effective therapy, numerous approaches are being investigated to mitigate intestinal damage, many of which aim at targeting the I/R-induced inflammatory response (Bertoni et al., 2018). Cyclooxygenase-2 (COX-2), the inducible form of the COX enzyme, plays a key role in inflammation by producing proinflammatory prostaglandins from arachidonic acid (Ricciotti and FitzGerald, 2011). Gene and protein expression of COX-2, but not the constitutively active COX-1 isoform, increase rapidly in response to intestinal I/R (Blikslager et al., 2002; Sato et al., 2005; Moses et al., 2009), suggesting that COX-2 is a potential target for the treatment of intestinal I/R injury. Indeed, based on the majority of available, albeit limited, studies non-selective (Schildberg et al., 2016; Ucar et al., 2020) and selective COX-2 inhibitors (Kawata et al., 2003; Arumugam et al., 2003; Sato et al., 2005; Moses et al., 2009; Gugliandolo et al., 2020; Li and Zheng, 2021) provide varying degrees of protection against I/R-associated small intestinal inflammation and tissue damage. In addition, besides having the potential to mitigate intestinal injury triggered by local I/R, long-term inhibition of COX-2 may even reduce intestinal damage evoked by I/R of remote organs (László et al., 2020). The chronic use of selective COX-2 inhibitors, however, is associated with an increased risk for cardiovascular events (Funk and FitzGerald, 2007), therefore the potential benefits of these drugs against I/R injury could be exploited mainly by using them in the short-term, for example, to prevent intestinal damage caused by a cardiopulmonary bypass or small bowel transplantation (Schildberg et al., 2016).

Interestingly, the protective effect of COX-2 inhibitors against intestinal I/R injury is in sharp contrast to their pro-inflammatory and injury-promoting effects observed in gastric I/R injury. Several studies have shown unequivocally that COX-2-derived prostaglandins contribute to the maintenance of gastric mucosal integrity during I/R, and selective COX-2 inhibitors aggravate mucosal damage and delay the healing of gastric lesions (Brzozowski et al., 1999; Maricic et al., 1999; Hiratsuka et al., 2005; Kotani et al., 2006). Although the exact mechanisms underlying the different outcomes of COX-2 inhibition in the upper and lower gastrointestinal tract are still unclear, there is some evidence that prostaglandins generated by COX-2 may also be mucoprotective in the context of intestinal I/R injury. For example, it was shown that prostaglandins generated by both COX-1 and COX-2 are involved in the recovery of mucosal barrier in the ischemia-injured porcine ileum (Blikslager et al., 2002), and deletion of the COX-2 gene was associated with more severe injury and increased epithelial apoptosis after intestinal I/R in mice (Watanabe et al., 2012).

Identification of the exact role of COX-2 in I/R-induced gut injury is also complicated by the fact that COX-inhibitors may have additional, COX-independent effects as well, such as activation of PPAR-γ and heme oxygenase-1 (HO-1), or regulation of the phosphatidylinositol 3-kinase (PI3K)/Akt signaling (Tegeder et al., 2001; Little et al., 2007). In addition, COX-inhibitors can induce apoptosis via both COX-2-dependent and COX-2-independent mechanisms (Jana, 2008). Activation of these pathways can affect intestinal I/R injury by itself (Nakajima et al., 2001; Attuwaybi et al., 2004; Liu et al., 2015), and such off-target effects may contribute to or even cause the protective or harmful effect of certain COX-2 inhibitors (Sato et al., 2005).

Because of the limited and inconsistent data about the role of COX-2 in intestinal I/R injury, here we aimed to compare the effect of celecoxib and rofecoxib on the intestinal damage caused by mesenteric I/R in rats. Rofecoxib, a drug withdrawn from the market due to serious adverse cardiovascular effects (Baron et al., 2008), was chosen as a comparator because of its higher COX-2 selectivity (Warner et al., 1999) and different off-target profile compared to celecoxib (Tegeder et al., 2001; Little et al., 2007).

## 2 Materials and methods

### 2.1 Animals

Experiments were carried out on 8–12 week-old male Wistar rats weighing 250–350 g (Toxi-Coop Ltd., Budapest, Hungary). Animals were housed in a temperature (22°C ± 2°C)- and humidity-controlled room at a 12 h light/dark cycle. Food and water were available *ad libitum*. All efforts were made to minimize animal suffering and to reduce the number of animals used in the experiments. All procedures conformed to the Directive 2010/63/EU on European Convention for the protection of animals used for scientific purposes. The experiments were approved by the National Scientific Ethical Committee on Animal Experimentation and permitted by the government (Food Chain Safety and Animal Health Directorate of the Government Office for Pest County (PE/EA/1118–6/2020)).

### 2.2 Study design

**FIGURE 1:**
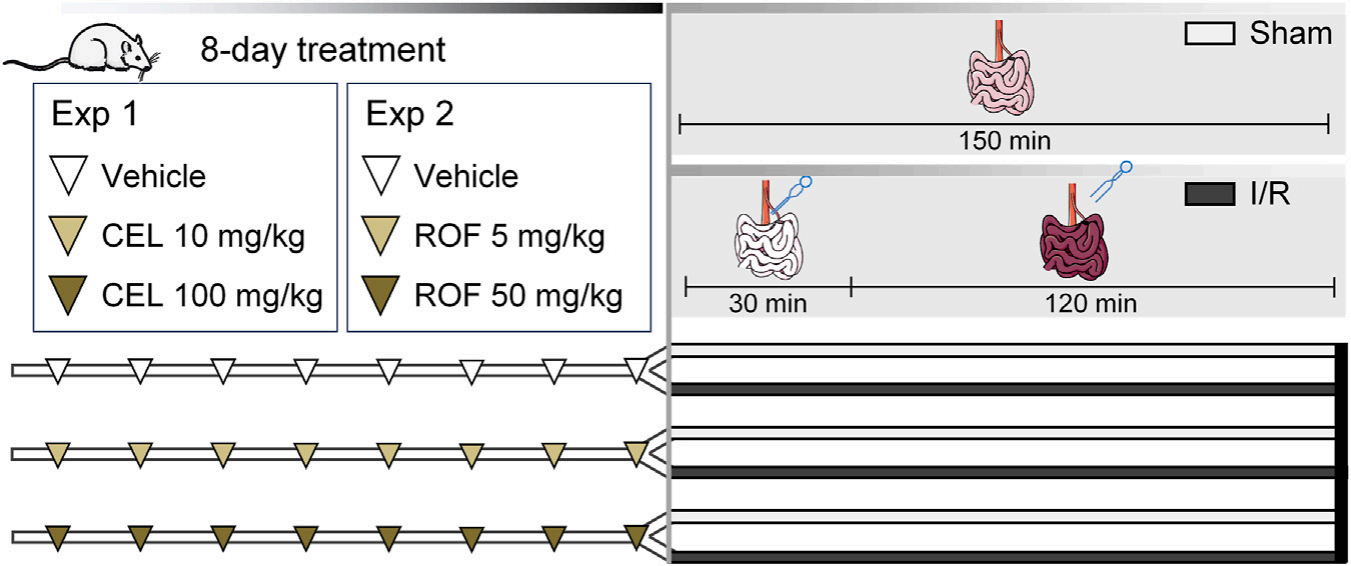
Experimental protocol. Male Wistar rats were treated with vehicle (1% hydroxyethylcellulose), celecoxib (CEL, 10 and 100 mg/kg), or rofecoxib (ROF, 5 and 50 mg/kg) for 8 days once daily. On the eighth day, 2 h after the last drug administration, rats were subjected to either sham operation or mesenteric ischemia/reperfusion (I/R). n = 7–8/group.

In the present study, two *in vivo* experiments were performed. In the first one, 48 rats were equally and randomly divided into three groups and were treated once daily for 8 days with either celecoxib (10 and 100 mg/kg) (Merck Millipore, Burlington MA, United States) or its vehicle (1% hydroxyethylcellulose) via gavage. The 10 mg/kg dose of celecoxib was reported to be selective for COX-2 over COX-1 (Gambero et al., 2005) and also our preliminary studies confirmed the high efficacy and COX-2 selectivity of celecoxib in this dose range (Supplementary Figure 1). The higher dose was chosen based on studies showing that celecoxib can also elicit COX-independent effects at this dose (Fan et al., 2011; Al-Rashed et al., 2018). On the eighth day, 2 h after the final drug administration, animals in all three groups were divided further into 2-2 groups, with eight rats in each group, and were anesthetized by intraperitoneal injection of pentobarbital (60 mg/kg). After upper median laparotomy animals were subjected to either sham operation, in which the superior mesenteric artery (SMA) was isolated but not occluded, or to intestinal I/R injury by occluding SMA for 30 min and then allowing blood reperfusion for 120 min (Figure 1). This I/R injury protocol was chosen based on the results of our previous studies aiming to achieve sufficient intestinal injury and inflammation without significant mortality (Supplementary Figure 2).

During surgery, the body temperature of animals was maintained at 37°C with a heating pad, and the depth of anesthesia was monitored by periodically assessing the pedal reflexes. At the end of reperfusion, the rats were euthanized and their small intestines were excised. The mucosa of the small intestine was flushed with cold saline, and three samples, each 1–2 cm long, were taken from the same part of the distal jejunum, 13–15 cm from the ileocecal junction. The first two specimens were snap-frozen in liquid nitrogen and stored at −80°C for measurement of protein and gene expressions, whereas the third one was fixed in 10% formalin for histological analysis.

In the second experiment essentially the same protocol was used, except that rats were treated with rofecoxib (MedChemExpress, Sollentuna, Sweden) instead of celecoxib (Figure 1). The applied doses of rofecoxib were chosen based on previous studies of other groups and ours (Gambero et al., 2005; Lázár et al., 2019).

### 2.3 Western blot

Distal jejunal tissues were homogenized with a TissueLyser (Qiagen, Venlo, Netherlands) in lysis buffer supplemented with a protease inhibitor cocktail (cOmplete ULTRA Tablets, Roche, Basel, Switzerland) and PMSF (Sigma, St. Louis, MO, United States). The homogenates were centrifuged twice at 1500 × *g* and 4°C for 15 min and the supernatants were collected, their protein concentration was measured by the bicinchoninic acid assay (BCA, Thermo Fisher Scientific, Waltham, MA, United States). Equal amount of protein (20 μg) was mixed with Pierce Lane Marker reducing sample buffer (Thermo Fisher Scientific, Waltham, MA, United States), and loaded and separated in a 4%–20% precast Tris-glycine SDS polyacrilamide gel (Bio-Rad, Hercules, CA, United States). Proteins were transferred electrophoretically onto a polyvinylidene difluoride membrane (Bio-Rad, Hercules, CA, United States) at 200 mA overnight. Membranes were blocked with either 5% nonfat dry milk (Bio-Rad, Hercules, CA, United States) or 5% bovine serum albumin (BSA, a9647, Merck Millipore, Burlington, MA, United States) in Tris-buffered saline containing 0.05% Tween-20 (0.05% TBS-T; Sigma, St. Louis, MO, United States) at room temperature for 2 h. Membranes were incubated with primary antibodies against COX-2 (#12282, 1:500, Cell Signaling Technology, Danvers, MA, United States), myeloperoxidase (MPO, AF3667, 1:1,000, R&D Systems, Minneapolis, MN, United States), pentraxin 3 (PTX3, ab125007, 1:1,000, Abcam, Cambridge, United Kingdom), claudin-1 (ab15098, 1:1,000, Abcam, Cambridge, United Kingdom), occludin (ABT146, 1:1,000, Merck Millipore, Burlington, MA, United States), phospho-Akt (#9271, 1:1,000, 1,000, Cell Signaling Technology, Danvers, MA, United States) and Akt (/#9272, 1:1,000, Cell Signaling Technology, Danvers, MA, United States) overnight at 4°C, followed by 2 h incubation at room temperature with an appropriate HRP-linked secondary antibody. GAPDH (D16H11, 1:1,000, Cell Signaling Technology, Danvers, MA, United States) and Akt (in the case of phospho-Akt) were used as loading controls. Membranes were trimmed before the antibody treatment if the bands of interest were far apart. At least two repetitions were performed for each experiment. Signals were detected with a chemiluminescence kit (Bio-Rad, Hercules, CA, United States) by Chemidoc XRS+ (Bio-Rad, Hercules, CA, United States). Relative protein levels were quantified by densitometric analysis using Image Lab Software version 6.1.0 (Bio-Rad, Hercules, CA, United States). The volume (intensity) of each band was quantified and normalized to the intensity of the respective control (GAPDH or Akt).

### 2.4 Histological analysis

Distal small intestinal samples were excised and prepared using the Swiss-roll technique, then were fixed in 10% formalin, embedded in paraffin, sectioned (4 µm), and stained with hematoxylin and eosin. Histological injury was graded on an eight-point scale, ranging from 0 (normal mucosa) to 8 (transmural infarction), in blinded fashion by two histopathologists according to the Chiu/Park classification (Chiu et al., 1970; Park et al., 1990), a widely used and highly reliable scoring system for grading intestinal I/R injury (Quaedackers et al., 2000). Representative pictures were captured by Eclipse E200 microscope and scanned by a Pannoramic 1000 Digital Slide Scanner.

### 2.5 qRT-PCR

Total RNA was obtained from 10 to 30 mg of small intestine tissue using the QIAzol extraction method (Qiagen, Hilden, Germany). RNA concentration was measured with a Nanophotometer (Implen GmbH, Munich, Germany). Reverse transcription was performed from 1 μg of total RNA with a Sensifast cDNA synthesis kit (Bioline, London, United Kingdom) according to the manufacturer’s protocol. Target genes were amplified using a LightCycler^®^ 480 II instrument (Roche, Germany) using the SensiFAST SYBR Green master mix (Bioline, United Kingdom). Expression levels were calculated with the 2^−ΔΔCT^ evaluation method and *Rpl13a* was used as a reference gene. Primers used for determination had the following sequences: *heme oxygenase-1 (HO-1)* forward AAG AGG CTA AGA CCG CCT TC, *HO-1* reverse GCA TAA ATT CCC ACT GCC AC (Accession number: NM_012580.2)*, peroxisome proliferator-activated receptor-γ (PPAR-γ)* forward CCC ACC AAC TTC GGA ATC AG, *PPAR-γ* reverse GGA ATG GGA GTG GTC ATC CA (Accession number: NM_013124), *interleukin 1β (IL-1β)* forward TGG CAA CTG TCC CTG AAC TC, *IL-1β* reverse GGG CTT GGA AGC AAT CCT TAA TC (Accession number: NM_031512.2), *interleukin-10 (IL-10)* forward GAA CCA CCC GGC ATC TAC TG, *IL-10* reverse AGG AGT TGC TCC CGT TAG C (Accession number: NM_012854.2), *B cell lymphoma-2 (Bcl-2)* forward TGA GTA CCT GAA CCG GCA TC, *Bcl-2* reverse TAT AGT TCC ACA AAG GCA TCC CAG (Accession number: NM_009741.5), *Bcl-2 associated X-protein (Bax)* forward AGT GTC TCC GGC GAA TTG G, *Bax* reverse CAC GTC AGC AAT CAT CCT CTG C (Accession number: NM_007524.4) and *Rpl13a* forward GGA TCC CTC CAC CCT ATG ACA, *Rpl13a* reverse CTG GTA CTT CCA CCC GAC CTC (Accession number: NM_173340.2). At least two repetitions were performed for each experiment.

### 2.6 COX enzyme activity assay

The total COX enzyme activity of homogenized intestinal samples (10 μL) was measured by a fluorescent COX-activity assay kit (700200, Cayman Chemical, Ann Arbor, MI, United States) according to the manufacturer’s instructions. Sample homogenization was performed as described in Section 2.4. The fluorescence (λ_excitation_ = 535 nm and λ_emission_ = 590 nm) was recorded at 5 min by a Varioskan™ LUX Multimode Microplate Reader (Thermo Fisher Scientific, Waltham, MA, United States). All samples were measured in duplicates. To assess the contribution of COX-1 and COX-2 isoforms to total COX enzyme activity, the highly selective COX-1 inhibitor SC-560 was added to separate sample aliquots (final well concentration: 3.47 μM). Enzyme activities were expressed as percentage of the mean activity of the vehicle-treated sham-operated groups.

### 2.7 Statistics

Data are expressed as mean + SEM. Statistical analysis of the data was performed with two-way ANOVA followed by Fisher’s LSD *post hoc* test, or with Kruskal-Wallis test and uncorrected Dunn’s *post hoc* test (in the case of histological scores). Outliers detected by Grubb’s test were excluded from the analyses. In all cases, a probability of p < 0.05 was considered statistically significant.

## 3 Results

### 3.1 Celecoxib, but not rofecoxib, reduced the severity of intestinal inflammation induced by mesenteric I/R

**FIGURE 2:**
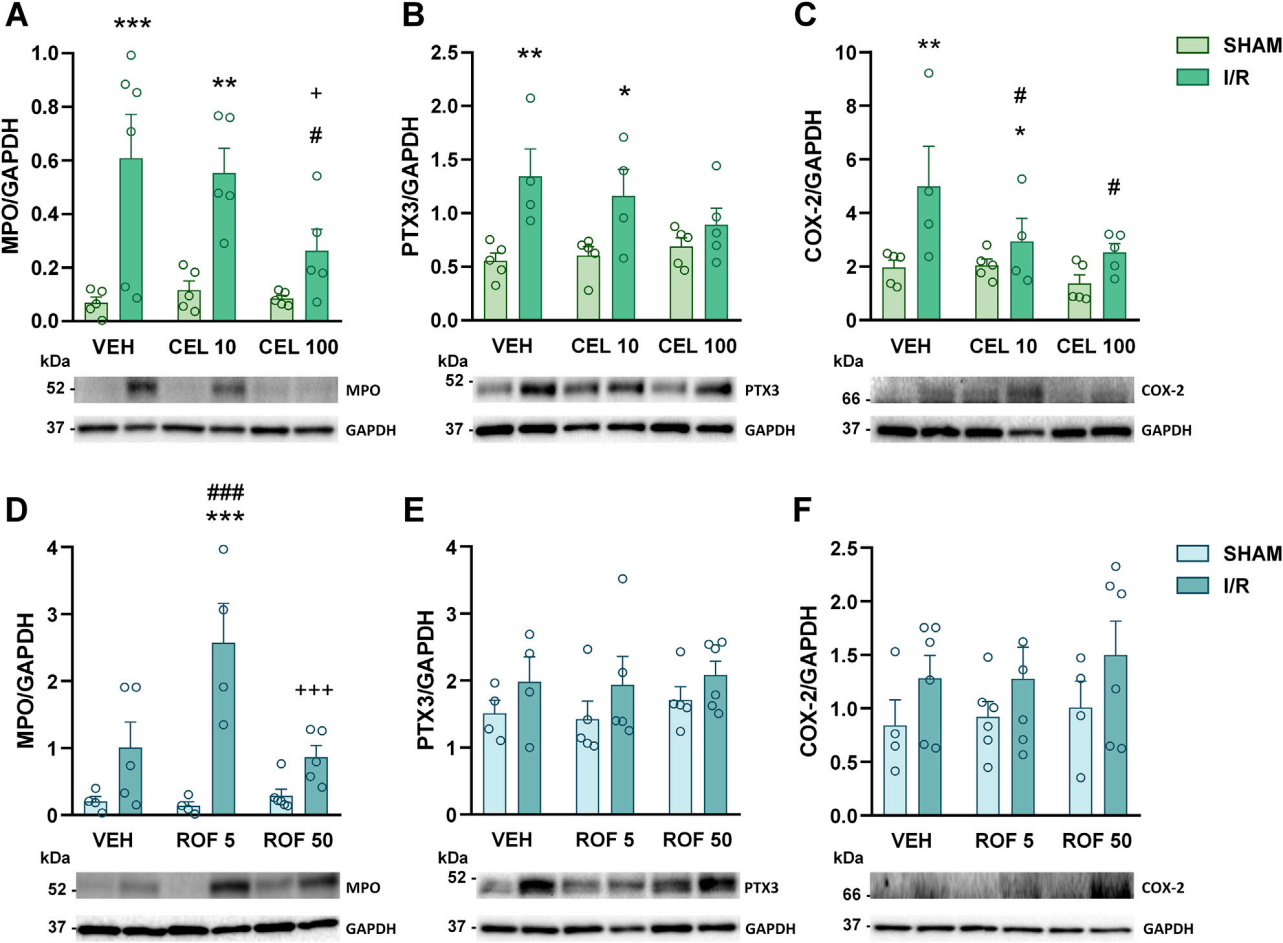
Small intestinal levels of MPO **(A, D)**, pentraxin 3 (PTX3, **(B, E)** and cyclooxygenase-2 proteins (COX-2, **(C, F)** in rats treated with vehicle (VEH), celecoxib (CEL, 10 and 100 mg/kg) or rofecoxib (ROF, 5 and 50 mg/kg) for 8 days and then subjected to sham operation or mesenteric I/R. Circles represent the data of each rat, bars indicate the mean + SEM. For statistical analysis two-way ANOVA was used, followed by Fisher’s LSD *post hoc* test. n = 4–6/group, *p < 0.05, **p < 0.01, ***p < 0.001 vs. respective SHAM, ^#^p < 0.05, ^##^p < 0.01, ^###^p < 0.001 vs. VEH I/R, ^+^p < 0.05 vs. CEL 10 I/R, ^+++^p < 0.001 vs. ROF 5 I/R.

There were no macroscopic changes in the small intestines of sham-operated rats, whereas the intestines of mesenteric I/R-exposed rats were livid and edematous with hemorrhages. We first aimed to assess the severity of intestinal inflammation caused by mesenteric I/R in control (vehicle-treated) and COX-2 inhibitor-treated animals. Because recruitment and activation of neutrophils is a key component of intestinal I/R-induced inflammation and mucosal injury (Kurtel et al., 1991), first we measured the tissue levels of the neutrophil marker MPO by Western blotting. Intestinal I/R was associated with upregulation of MPO, which was partially prevented by celecoxib treatment, although significant reduction of MPO was achieved only in animals treated with the higher dose of celecoxib. In contrast, rofecoxib failed to reduce the MPO-increasing effect of I/R, in fact, at the lower dose it even promoted it (Figures 2A, D).

Next, we measured the protein levels of pentraxin 3 (PTX3), a member of the long pentraxin family, which is released by a variety of cell types in response to proinflammatory cytokines and other inflammatory signals and has an important role in the regulation of I/R-induced intestinal inflammation (Souza et al., 2009). Of the treatments tested, only the highest dose of celecoxib prevented the I/R-induced elevation of PTX3 (Figures 2B, E).

**FIGURE 3:**
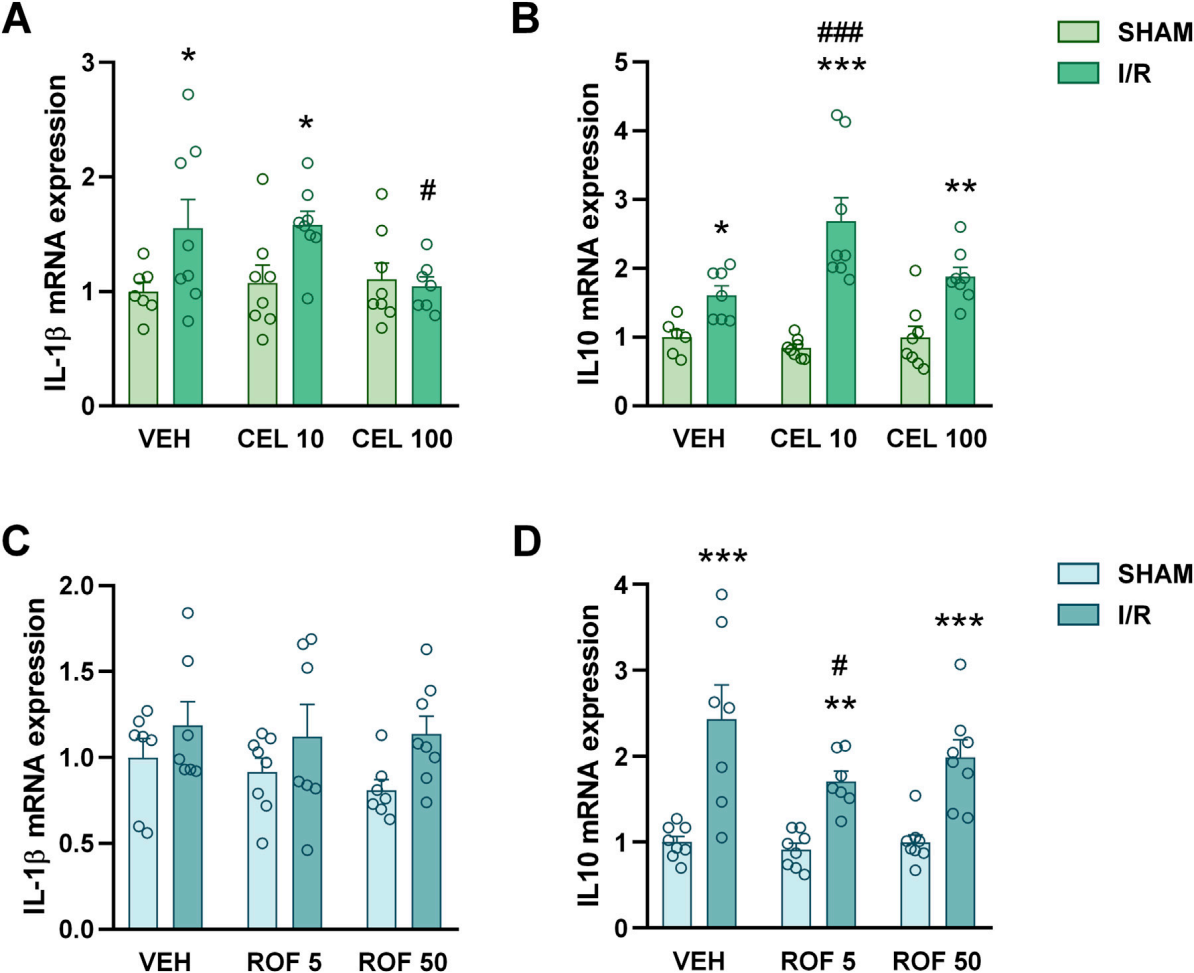
Small intestinal mRNA levels of interleukin-1β (IL-1β, **(A, C)** and interleukin 10 (IL-10, **(B, D)** in rats treated with vehicle (VEH), celecoxib (CEL, 10 and 100 mg/kg) or rofecoxib (ROF, 5 and 50 mg/kg) for 8 days and then subjected to sham operation or mesenteric I/R. Circles represent the data of each rat, bars indicate the mean + SEM. For statistical analysis two-way ANOVA was used, followed by Fisher’s LSD *post hoc* test. n = 6–8/group, *p < 0.05, **p < 0.01, ***p < 0.001 vs. respective SHAM, ^#^p < 0.05, ^###^p < 0.001 vs. VEH I/R.

Treatment with celecoxib, but not with rofecoxib, also reduced the intestinal upregulation of COX-2 protein in animals exposed to mesenteric I/R (Figures 2C, F), as well as the elevation of IL-1β mRNA (Figures 3A, C), a well-established inducer of COX-2 expression (Tsatsanis et al., 2006). We also measured the mRNA expression of the anti-inflammatory cytokine IL-10. Although celecoxib increased, whereas rofecoxib decreased the level of IL-10, the effect was caused only by the lower doses of drugs (Figures 3B, D).

Collectively, of the two drugs tested, only celecoxib reduced the severity of intestinal inflammation evoked by mesenteric I/R, and even the higher dose of celecoxib caused only a partial reduction of the inflammatory mediators measured. There were no signs of inflammation in any of the sham-operated rats, suggesting that 8-day treatments with celecoxib and rofecoxib had no significant effect on intestinal mucosal integrity.

### 3.2 Neither celecoxib nor rofecoxib mitigated the histological injury caused by mesenteric I/R, but high-dose celecoxib prevented the disruption of tight junction proteins

**FIGURE 4:**
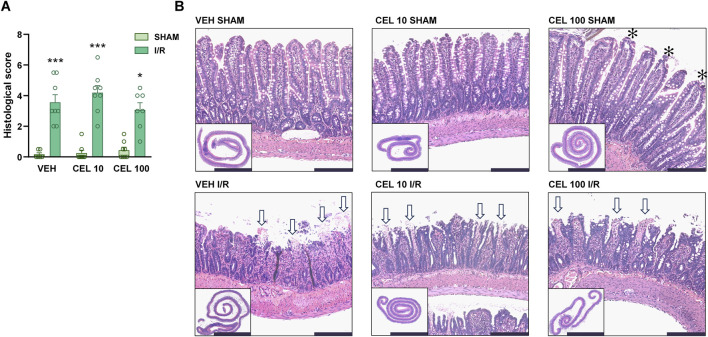
The effect of vehicle (VEH) and celecoxib (CEL, 10 and 100 mg/kg) on the histomorphology of the small intestinal mucosa in sham-operated and I/R-exposed rats. **(A)** Histological scores. Circles represent the data of each rat, bars indicate the mean + SEM. For statistical analysis, Kruskal-Wallis test was performed, followed by uncorrected Dunn’s test. n = 7–8/group, *p < 0.05, ***p < 0.001 vs. respective SHAM. **(B)** Representative histological images of the small intestines of VEH- and CEL-treated rats. Haematoxylin and eosin staining, low magnification scale bar (lower left images): 5 mm, high magnification scale bar: 200 μm. White arrows mark denuded villi with lamina propria and capillaries exposed, asterisks show moderate lifting of the epithelial layer from the lamina propria (Gruenhagen’s space).

**FIGURE 5:**
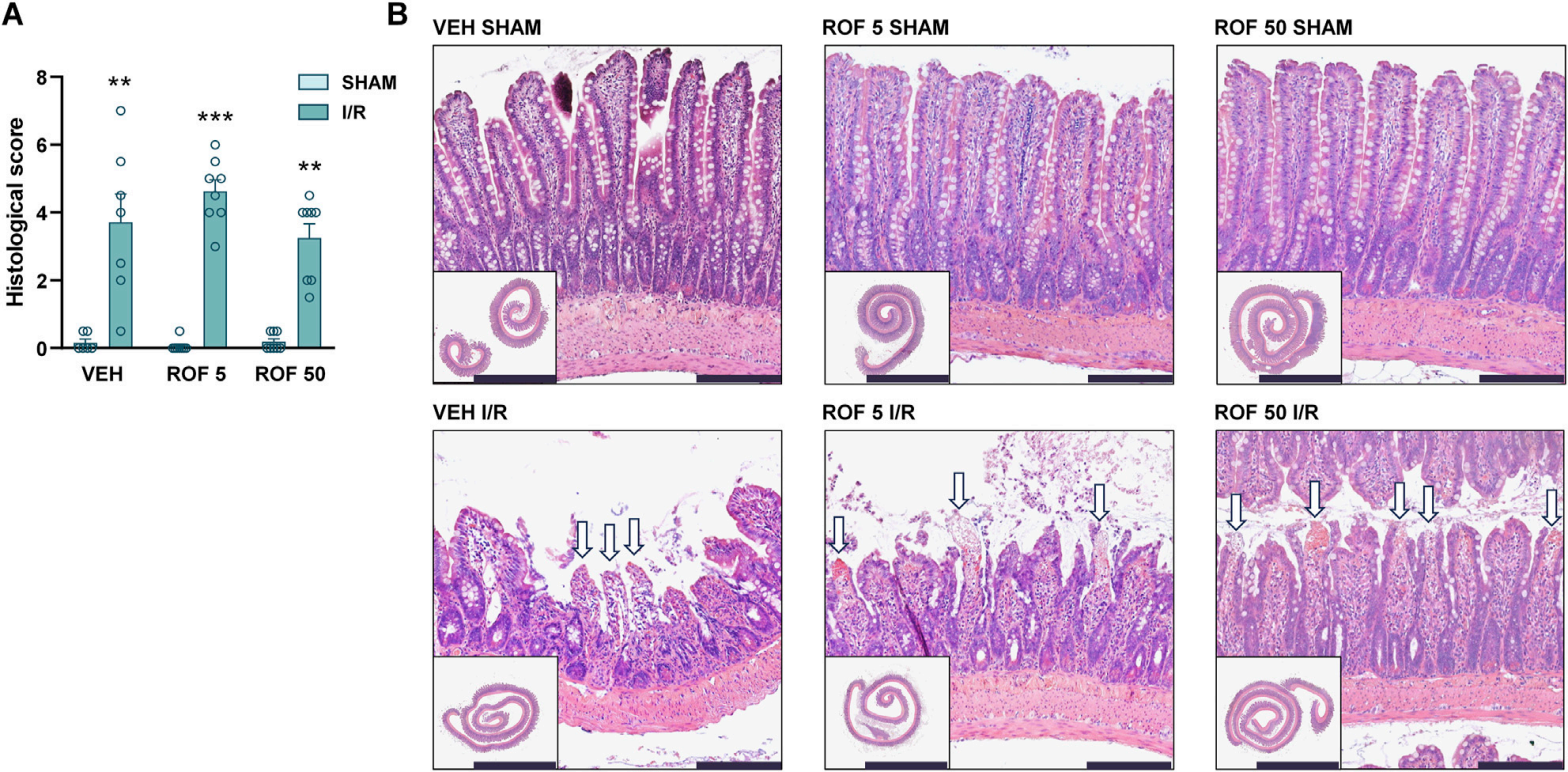
The effect of vehicle (VEH) and rofecoxib (ROF, 5 and 50 mg/kg) on the histomorphology of the small intestinal mucosa in sham-operated and I/R-exposed rats. **(A)** Histological scores. Circles represent the data of each rat, bars indicate the mean + SEM. For statistical analysis, Kruskal-Wallis test was performed, followed by uncorrected Dunn’s test. n = 7–8/group, **p < 0.01, ***p < 0.001 vs. respective SHAM. **(B)** Representative histological images of the small intestines of VEH- and ROF-treated rats. Haematoxylin and eosin staining, low magnification scale bar (lower left images): 5 mm, high magnification scale bar: 200 μm. White arrows mark denuded villi with lamina propria and capillaries exposed.

Next, we set out to determine whether treatment with celecoxib and rofecoxib can attenuate mucosal injury caused by intestinal I/R. There was no significant mucosal damage in any of the sham-operated rats, although, in some animals treated with the higher dose of celecoxib, we observed some mild changes, such as slightly dilated villi and subepithelial Gruenhagen’s space (Figures 4, 5). Mesenteric I/R induced various morphological alterations ranging from pathological lifting or destruction of the epithelium to more severe damage of the villi and sometimes even the crypts. Interestingly, celecoxib treatment had no effect on I/R-induced histological injury at the tested doses, despite reducing mucosal inflammation. Similarly, neither dose of rofecoxib affected the I/R-provoked histological changes in the mucosa (Figures 4, 5).

**FIGURE 6:**
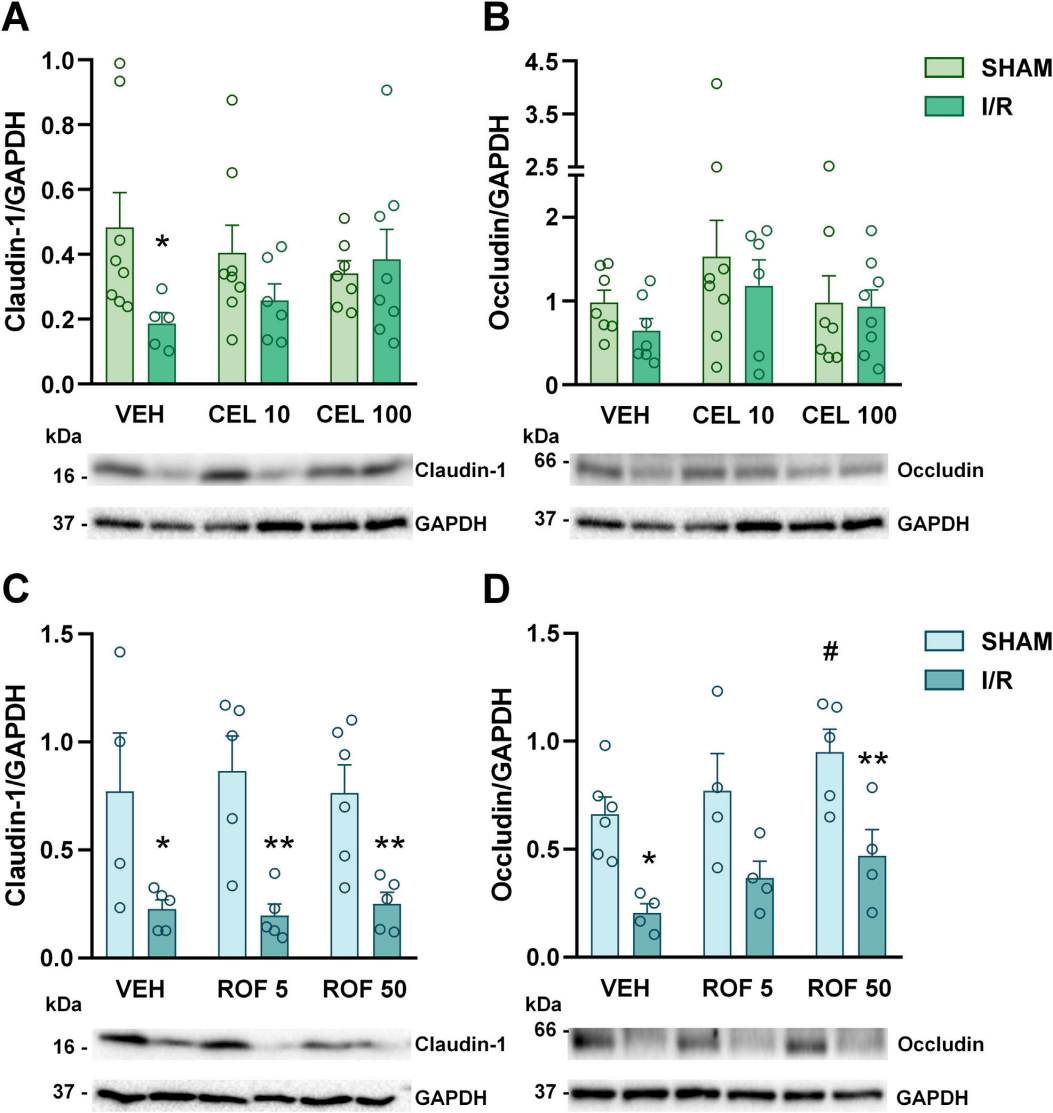
Small intestinal expressions of claudin-1 **(A, C)** and occludin proteins **(B, D)** in rats treated with vehicle (VEH), celecoxib (CEL, 10 and 100 mg/kg) or rofecoxib (ROF, 5 and 50 mg/kg) for 8 days and then subjected to sham operation or mesenteric I/R. Representative bands of claudin-1 and occludin in **(A, B)** are derived from the same animals, therefore images of GAPDH proteins are identical. Circles represent the data of each rat, bars indicate the mean + SEM. For statistical analysis two-way ANOVA was used, followed by Fisher’s LSD *post hoc* test. n = 4–8/group, *p < 0.05, **p < 0.01 vs. respective SHAM, ^#^p < 0.05 vs. VEH SHAM.

It is well-established that I/R-induced mucosal damage is associated with the disruption of tight junction proteins, such as claudin-1 and occludin (Li et al., 2009), therefore, we also assessed their expression by Western blotting. The expression of claudin-1 was significantly reduced in response to I/R in both cohorts, and this effect was prevented by the higher dose of celecoxib, but not by rofecoxib (Figures 6A, C). The measurement of occludin yielded essentially similar results, although in this case, the reduction in occludin expression caused by I/R was not statistically significant in the first experiment (Figures 6B, D).

Taken together, celecoxib had no significant impact on the histological signs of I/R-induced mucosal damage but at the higher dose prevented the loss of tight junction proteins, whereas rofecoxib had no effect on any of them.

### 3.3 Celecoxib increased I/R-induced intestinal apoptosis, whereas rofecoxib reduced the I/R-induced phosphorylation of Akt

Because celecoxib and rofecoxib had different effects on intestinal inflammation and tight junction proteins despite both being able to almost completely inhibit COX-2 activity at the doses used (Fornai et al., 2014; Lázár et al., 2019), we hypothesized that the observed differences may be independent of COX-inhibition and assessed the levels of some known off-targets of COX-2 inhibitors.

**FIGURE 7:**
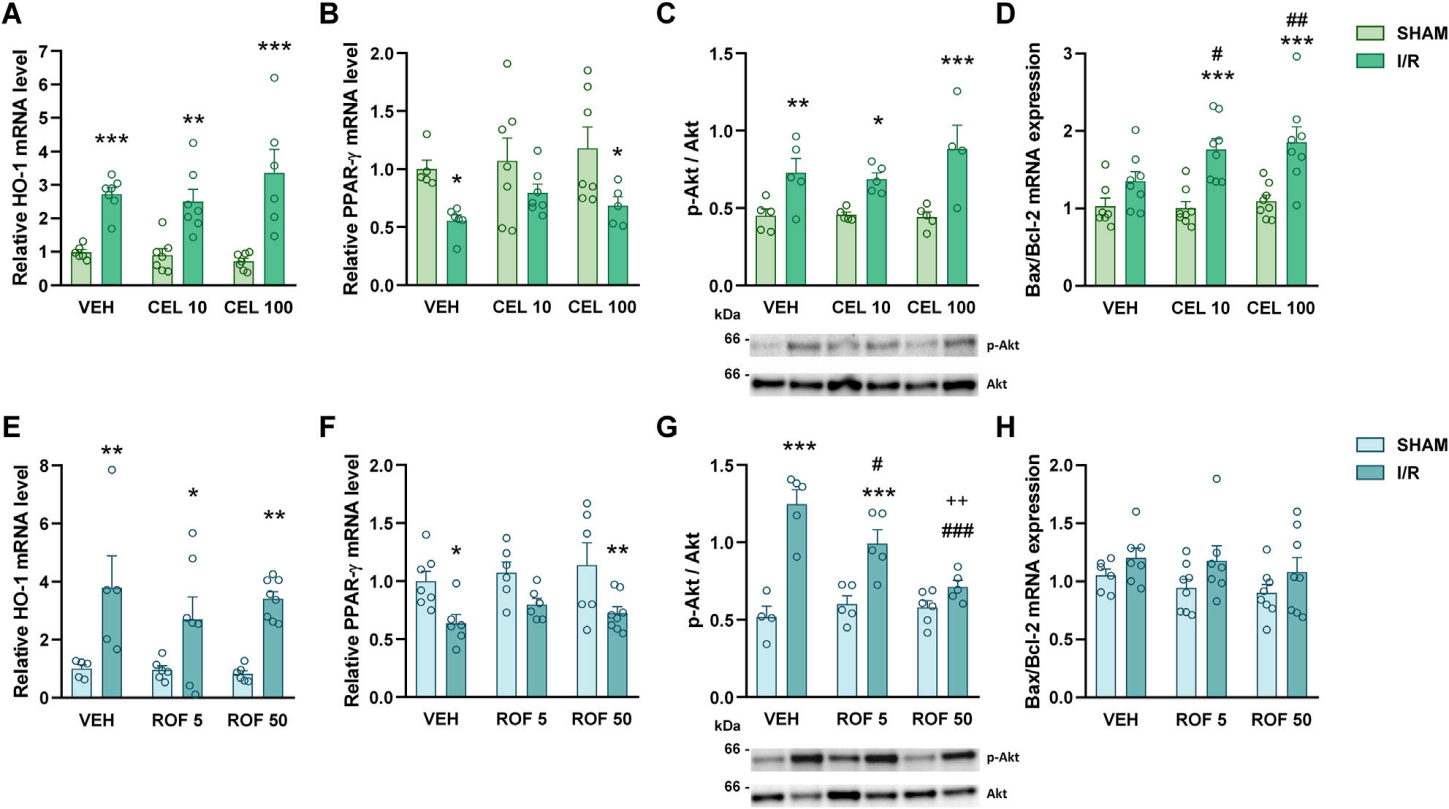
Gene expression of heme oxygenase-1 (HO-1, **(A, E)** and peroxisome proliferator-activated receptor-γ (PPAR-γ, **(B, F)** and the ratio of phosphorylated to total Akt protein (p-Alt/Akt), **(C, G)** and Bax to Bcl-2 mRNA **(D, H)** in the small intestine of rats treated with vehicle (VEH), celecoxib (CEL, 10 and 100 mg/kg) or rofecoxib (ROF, 5 and 50 mg/kg) for 8 days and then subjected to sham operation or mesenteric I/R. Circles represent the data of each rat, bars indicate the mean + SEM. For statistical analysis two-way ANOVA was used, followed by Fisher’s LSD *post hoc* test. n = 4–8/group, *p < 0.05, **p < 0.01, ***p < 0.001 vs. respective SHAM, ^#^p < 0.05, ^##^p < 0.01, ^###^p < 0.001 vs. VEH I/R, ^++^p < 0.01 vs. ROF 5 I/R.

First, we measured the gene expression of the stress-inducible enzyme HO-1, which was shown to be induced by celecoxib, but not by rofecoxib (Hamdulay et al., 2010), and is upregulated in, and protects against intestinal I/R (Attuwaybi et al., 2004). Although our results confirmed that I/R increases the expression of HO-1, there was no difference between the HO-1 levels of vehicle- and drug-treated animals, neither in the sham-operated nor in the I/R-injured groups (Figures 7A, E). Next, we assessed the expression of PPAR-γ, a nuclear receptor mediating anti-inflammatory effects in the context of intestinal I/R injury (Nakajima et al., 2001) and being activated by several COX-inhibitors, including celecoxib and rofecoxib (Konturek et al., 2003; López-Parra et al., 2005). Mesenteric I/R caused a significant drop in intestinal PPAR-γ expression, but neither this nor the basal expression of PPAR-γ in sham-operated rats was affected by celecoxib or rofecoxib significantly (Figures 7B, F). Because celecoxib can activate the PI3K/Akt pathway (Hou et al., 2005), which otherwise was shown to reduce inflammation and barrier damage in intestinal I/R injury (Huang et al., 2011), we also determined the phosphorylation of Akt. In contrast to what was expected, celecoxib treatment had no effect on phospho-Akt levels in either the sham-operated or the I/R-injured groups, whereas rofecoxib prevented the I/R-induced elevation of phospho-Akt in a dose-dependent manner (Figures 7C, G). Finally, because apoptosis is a major mode of epithelial cell death caused by I/R injury (Ikeda et al., 1998), and celecoxib was shown to increase epithelial apoptosis (Kazanov et al., 2004), we aimed to determine whether the inability of celecoxib to decrease I/R-induced mucosal injury despite inhibiting inflammation may be related to increased apoptosis. Therefore, we measured the gene expression of Bax and Bcl-2, and calculated the Bax/Bcl-2 ratio, an important marker of apoptosis (Oltvai et al., 1993). The ratio of Bax to Bcl-2 showed a modest, non-significant elevation in response to I/R, which was enhanced by celecoxib, but was not affected by rofecoxib (Figures 7D, H).

Collectively, celecoxib increased I/R-induced apoptosis, whereas rofecoxib reduced the I/R-induced elevation of phospho-Akt.

### 3.4 High-dose celecoxib, but not rofecoxib, reduced the activity of COX-1 in the small intestine

Finally, because not only COX-2 but also COX-1 can contribute to prostanoid release during inflammation (Ricciotti and FitzGerald, 2011), we addressed whether the difference between the effect of high-dose celecoxib and rofecoxib on intestinal I/R inflammation is due to their different effect on COX-1 activity. To this end, we measured total COX activities in the small intestines of celecoxib- and rofecoxib-treated animals by an assay kit, and assessed the contribution of COX-1 to total COX activity by measuring COX activities of the same samples in both the presence and absence of SC-560, a highly selective COX-1 inhibitor.

**FIGURE 8:**
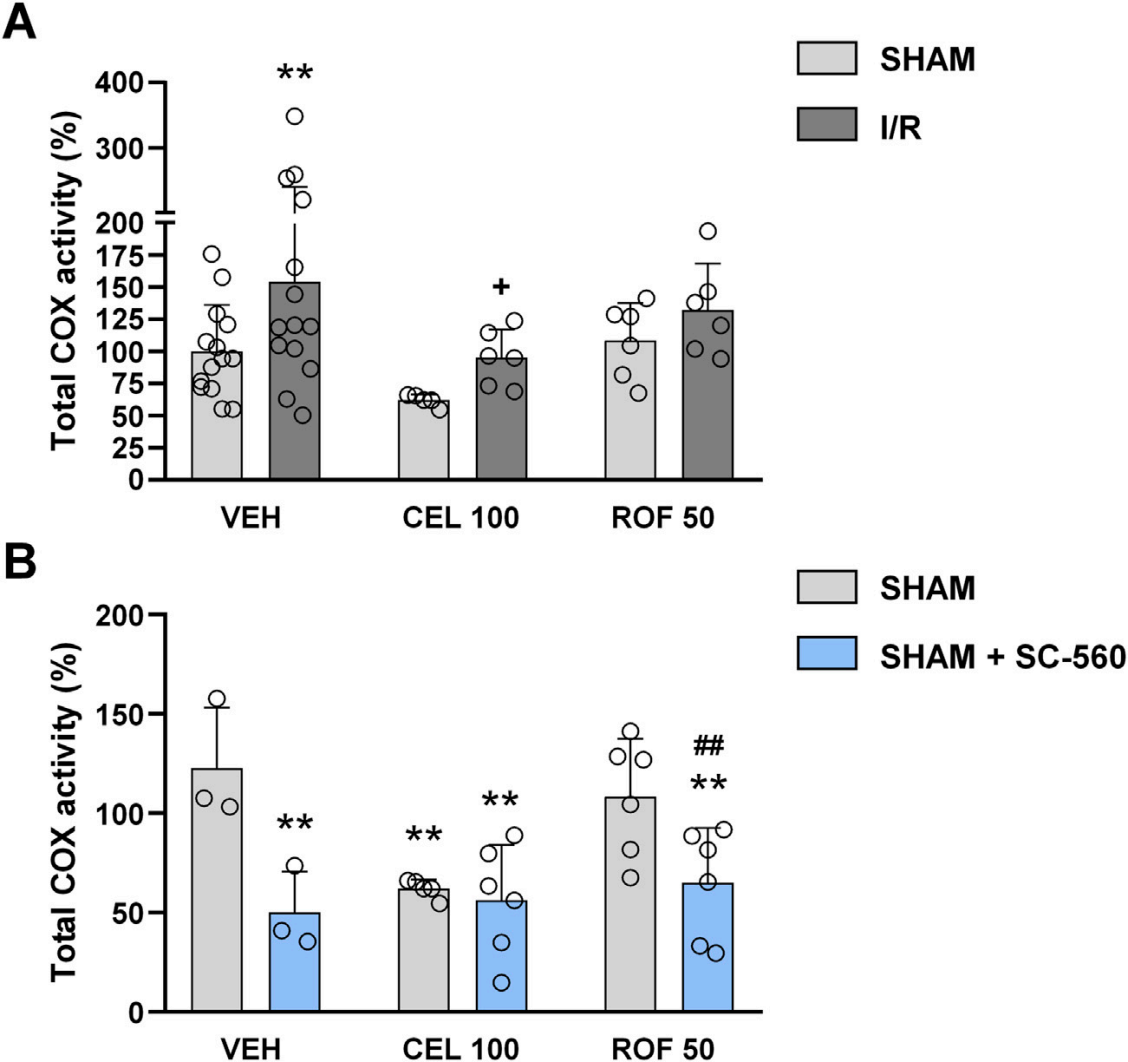
Total cyclooxygenase (COX) activity in the small intestine of rats treated with vehicle (VEH), celecoxib (CEL, 100 mg/kg), or rofecoxib (ROF, 50 mg/kg) for 8 days and then subjected to sham operation or mesenteric I/R **(A)**. COX activity of some samples was also measured in the presence of SC-560, a highly selective COX-1 inhibitor, to assess the contribution of COX-1 and COX-2 to total COX activity **(B)**. Circles represent the data of each rat, bars indicate the mean + SEM. For statistical analysis two-way ANOVA was used, followed by Fisher’s LSD *post hoc* test. n = 3–14/group, **p < 0.05 vs. VEH SHAM, ^+^p < 0.05 vs. VEH I/R, ^##^p < 0.01 vs. ROF 50 SHAM.

Mesenteric I/R increased intestinal total COX activity significantly in vehicle-treated rats (VEH SHAM vs. VEH I/R, p = 0.009) (Figure 8A). In contrast, COX activity in celecoxib- and rofecoxib-treated I/R-exposed animals was comparable to that of the vehicle-treated sham-operated group (VEH SHAM vs. CEL I/R and ROF I/R, p = 0.85 and p = 0.21, respectively), indicating that both celecoxib- and rofecoxib treatment could prevent the I/R-evoked elevation of COX-activity. However, celecoxib, but not rofecoxib, also reduced total COX activity in sham-operated animals (VEH SHAM vs. CEL SHAM, p = 0.003) (Figure 8B). In addition, spiking the samples with the COX-1 inhibitor SC-560 reduced total COX activity in both vehicle- and rofecoxib-treated sham-operated rats (VEH SHAM vs. VEH SHAM + SC 560, p = 0.002, ROF SHAM vs. ROF SHAM + SC-560, p = 0.006), but it did not reduce further the COX activity in celecoxib-treated sham-operated animals.

These results suggest that high-dose celecoxib reduced COX-1 activity in the small intestine, whereas rofecoxib remained selective for COX-2 at its higher dose.

## 4 Discussion

Here we show that two selective COX-2 inhibitors, celecoxib and rofecoxib, have different effects on mesenteric I/R-induced small intestinal inflammation in rats. Although neither drug could mitigate I/R-provoked histological injury, celecoxib, but not rofecoxib, reduced the severity of tissue inflammation and prevented the disruption of tight junction proteins. Significant inhibition of inflammation, however, was achieved mainly by using celecoxib at a high dose, which has already reduced COX-1 activity in the intestine. Celecoxib and rofecoxib had no significant effect on the basal expression of any of the off-targets tested, but celecoxib increased I/R-induced apoptosis, whereas rofecoxib reduced the I/R-induced phosphorylation of Akt. Our results suggest that selective COX-2 inhibition is not sufficient to prevent I/R-induced intestinal inflammation, because also COX-1 is likely to contribute to it, and even inhibition of inflammation by high-dose celecoxib cannot reduce I/R-induced mucosal damage.

Although the importance of COX-2-generated prostaglandins in inflammation is well recognized and intestinal I/R is associated with rapid upregulation of COX-2 (Blikslager et al., 2002; Moses et al., 2009), it is still a question of debate whether selective inhibition of COX-2 mitigates I/R-induced gut injury. In most animal studies addressing this question different COX-2 inhibitors have been used, and although a beneficial effect of these drugs was suggested in general, the results are far from convincing. For example, the selective COX-2 inhibitors FK3311 and NS-398 both reduced intestinal I/R injury (Kawata et al., 2003; Sato et al., 2005; Moses et al., 2009), although the protective effect of the latter compound was also shown to depend on the sex of animals used (Wu et al., 2020). In contrast, celecoxib and firocoxib afforded only moderate protection in rats (Arumugam et al., 2003; Gugliandolo et al., 2020), whereas the use of parecoxib resulted in different outcomes (Schildberg et al., 2016; Li and Zheng, 2021). Although these discrepancies may simply reflect methodologic heterogeneity across studies, such as differences in experimental animals and I/R protocols, they may also arise from differences in the pharmacological profile of COX-2 inhibitors. Our present findings that celecoxib, but not rofecoxib, ameliorated I/R-induced intestinal inflammation support the assumption that COX-2 inhibitors have different efficacy against intestinal I/R injury.

In the present study, celecoxib given at a dose sufficient to inhibit COX-2 activity selectively and almost completely (10 mg/kg) (Gambero et al., 2005; Supplementary Figure S2), was only marginally effective in preventing I/R-induced intestinal inflammation and loss of tight junction proteins and had no significant impact on the extent of histological injury. These findings are partly in line with those of a previous study showing that celecoxib at the same dose induced only partial protection against intestinal injury caused by a similar protocol in female rats (Arumugam et al., 2003). We found that rofecoxib, which is more selective for COX-2 than celecoxib (Warner et al., 1999), was even less effective, as it reduced neither intestinal inflammation nor mucosal injury, despite being used at highly effective doses (Gambero et al., 2005; Lázár et al., 2019). From these findings, it can be inferred that selective inhibition of COX-2 is not sufficient to mitigate I/R-induced small intestinal inflammation and damage, and the variable protection conferred by certain COX-2 inhibitors may also involve additional mechanisms.

In contrast to the lower dose, the 100 mg/kg dose of celecoxib reduced tissue inflammation significantly in mesenteric I/R-exposed rats and also prevented the loss of tight junction proteins. Previous studies assessing whole blood thromboxane synthesis or PGE_2_ levels in the dorsal skin or small intestine as indices for COX-1 activity suggested that this dose of celecoxib is still selective for COX-2 (Sigthorsson et al., 2002; Gambero et al., 2005; King et al., 2006). However, we found that high-dose celecoxib likely reduced to some extent intestinal COX-1 activity, because total COX activity in the small intestine of celecoxib-treated sham-operated rats was lower than in vehicle-treated rats and comparable to the activity in samples spiked with the COX-1 inhibitor SC-560. A mild inhibitory effect of high-dose celecoxib on COX-1 is also suggested by the results of the histological analysis showing some morphological alterations in the mucosa of sham-operated rats because development of mucosal injury requires the simultaneous inhibition of both COX-1 and COX-2 (Wallace et al., 2000; Tanaka et al., 2002). Nevertheless, insufficient selectivity of high-dose celecoxib for COX-2 may also account for its higher efficacy in our model. Namely, there is some evidence that also drugs with some preference for COX-1, such as flunixin and flurbiprofen, can reduce I/R-evoked intestinal inflammation (Arumugam et al., 2003; Ucar et al., 2020), which is in harmony with the fact that also COX-1-generated prostanoids can contribute to inflammation (Ricciotti and FitzGerald, 2011). Furthermore, piroxicam and meloxicam, drugs with lower selectivity for COX-2 were more effective in reducing intestinal I/R injury than parecoxib (Schildberg et al., 2016). Hence, inhibition of both COX isoforms is likely to be required to significantly reduce a full-fledged inflammation caused by intestinal I/R.

Despite reducing inflammation, a prominent component of the complex response of intestinal mucosa to reperfusion, high-dose celecoxib was ineffective in preventing I/R-induced mucosal injury. Nevertheless, this apparent contradiction may be explained by the dual proinflammatory and mucoprotective role of COX-derived prostaglandins in the gut (Wallace, 2008; Ricciotti and FitzGerald, 2011). Prostaglandins have been shown to prevent I/R-induced intestinal damage by reducing epithelial apoptosis (Topcu et al., 2007; Watanabe et al., 2012), whereas reduced COX-2 expression in mice lacking Toll-like receptor 4, MyD88, or lysophosphatidic acid type 2 receptor was associated with decreased inflammation but increased mucosal damage in different models of gut injury (Fukata et al., 2006; Watanabe et al., 2012; Hutka et al., 2024). The potential role of increased apoptosis in limiting the protective effect of celecoxib is also supported by the significantly higher Bax to Bcl-2 ratio in celecoxib-treated I/R-exposed rats. Interestingly, rofecoxib had no effect on this ratio, which is in line with previous findings that celecoxib has a larger apoptotic effect than rofecoxib (Kazanov et al., 2004; Winfield and Payton-Stewart, 2012), and also suggests that the pro-apoptotic effect of celecoxib in this model is independent of COX-2 inhibition.

Indeed, previous studies have identified several non-COX targets that can be affected by COX inhibitors, thereby influencing the efficacy or toxicity of these drugs (Tegeder et al., 2001; Little et al., 2007). Importantly, there are substantial differences in how different COX inhibitors modulate these pathways. For example, celecoxib, but not rofecoxib, was shown to increase the expression of HO-1, an enzyme with cytoprotective and anti-inflammatory properties, via generation of reactive oxygen species and activation of Akt (Hou et al., 2005; Hamdulay et al., 2010; Al-Rashed et al., 2018), whereas both celecoxib and rofecoxib increased the expression of PPAR-γ (Cui et al., 2005; Konturek et al., 2003). Activation of HO-1, Akt, and PPAR-γ can all mitigate intestinal I/R injury (Attuwaybi et al., 2004; Nakajima et al., 2001; Huang et al., 2011), and PPAR-γ is also involved in the protective effect of the selective COX-2 inhibitor NS-398 (Sato et al., 2005). Therefore, we sought to determine whether the observed difference between celecoxib and rofecoxib relies in part on activating these COX-independent mechanisms, but we did not find significant changes in the gene expression of HO-1 and PPAR-γ, or in the phosphorylation of Akt in the gut of celecoxib-treated animals. Interestingly, although in sham-operated rats rofecoxib had no influence on them either, it reduced the I/R-induced activation of Akt. Because the PI3K/Akt axis has a crucial role in the regulation of cell survival and inflammation (Hawkins and Stephens, 2015), it is plausible that the inability of rofecoxib to reduce inflammation was in part due to impaired activation of Akt after I/R, but this warrants further investigations.

In conclusion, our study shows that celecoxib is more effective than rofecoxib in preventing I/R-induced small intestinal inflammation and disruption of tight junction proteins in rats. Our findings that rofecoxib was ineffective at any of the tested doses and the beneficial effects of celecoxib were mainly observed at high, non-selective doses, suggest that selective COX-2 inhibition is not sufficient to mitigate I/R-induced intestinal inflammation because also COX-1 is likely to contribute to it. Importantly, despite inhibiting intestinal inflammation, even high-dose celecoxib did not affect the histomorphological changes of the I/R-exposed mucosa. These results suggest that selective COX-2 inhibitors have only limited therapeutic value in intestinal I/R injury.

## Data Availability

The original contributions presented in the study are included in the article/Supplementary Material, further inquiries can be directed to the corresponding author.

## Supplementary material

SUPPLEMENTARY FIGURE 1 The effect of celecoxib (CEL, 3 and 30 mg/kg) on the levels of COX-1-derived prostaglandin E2 (PGE2) in the gastric mucosa (left panel), and on COX-2-derived PGE2 in pouch exudate (right panel) in the carrageenan-airpouch model. Briefly, rats were treated intragastrically once daily with celecoxib or 1% hydroxyethylcellulose (vehicle) for 5 days in a volume of 0.33 mL/100 g. On the fifth day, 2 h after the final gavage, 2 mL of a 1% solution of lambda-carrageenan was injected into an air pouch, which was previously induced by injecting twice (on the first and third days of treatment) 10 mL sterile air subcutaneously into the intrascapular area of the rats under isoflurane anaesthesia. 3 h after the injection of carrageenan, the rats were anaesthetized and the pouch fluid was collected by lavage with 1 mL of cold heparin saline. Gastric mucosal samples were also collected, and PGE2 levels in both the pouch fluid and gastric mucosa were measured by ELISA (Cayman Chemical, Ann Arbor, MI, United States) according to the manufacturer’s protocol. The results are expressed as the mean + SEM percent of the control PGE2 levels measured in vehicle-treated rats, n = 5–6/group. ***p < 0.001 vs. control, for statistical analysis one-way ANOVA was used, followed by Fisher’s LSD *post hoc* test.

SUPPLEMENTARY FIGURE 2 Different I/R protocols were associated with different mortality **(A)** and small intestinal MPO levels **(B)**. Circles represent the data of each rat, bars indicate the mean + SEM. For statistical analysis one-way ANOVA was used, followed by Fisher’s LSD *post hoc* test. n = 3–6/group, ***p < 0.001 vs. respective SHAM.

## Abbreviations

Bax, Bcl-2 associated X-protein; Bcl-2, B cell lymphoma-2; COX, cyclooxygenase; HO-1, heme oxygenase-1; IL, interleukin; I/R, ischemia/reperfusion; MPO, myeloperoxidase; PTX3, pentraxin 3; PPAR-γ, peroxisome proliferator-activated receptor-γ; PI3K, phosphatidylinositol 3-kinase; SMA, superior mesenteric artery.
