# Supplementary figures and images for: Celecoxib and rofecoxib have different effects on small intestinal ischemia/reperfusion injury in rats
Source: Front Pharmacol. 2024 Nov 8;15:1468579. doi: 10.3389/fphar.2024.1468579 (PMC11582421; doi:10.3389/fphar.2024.1468579)

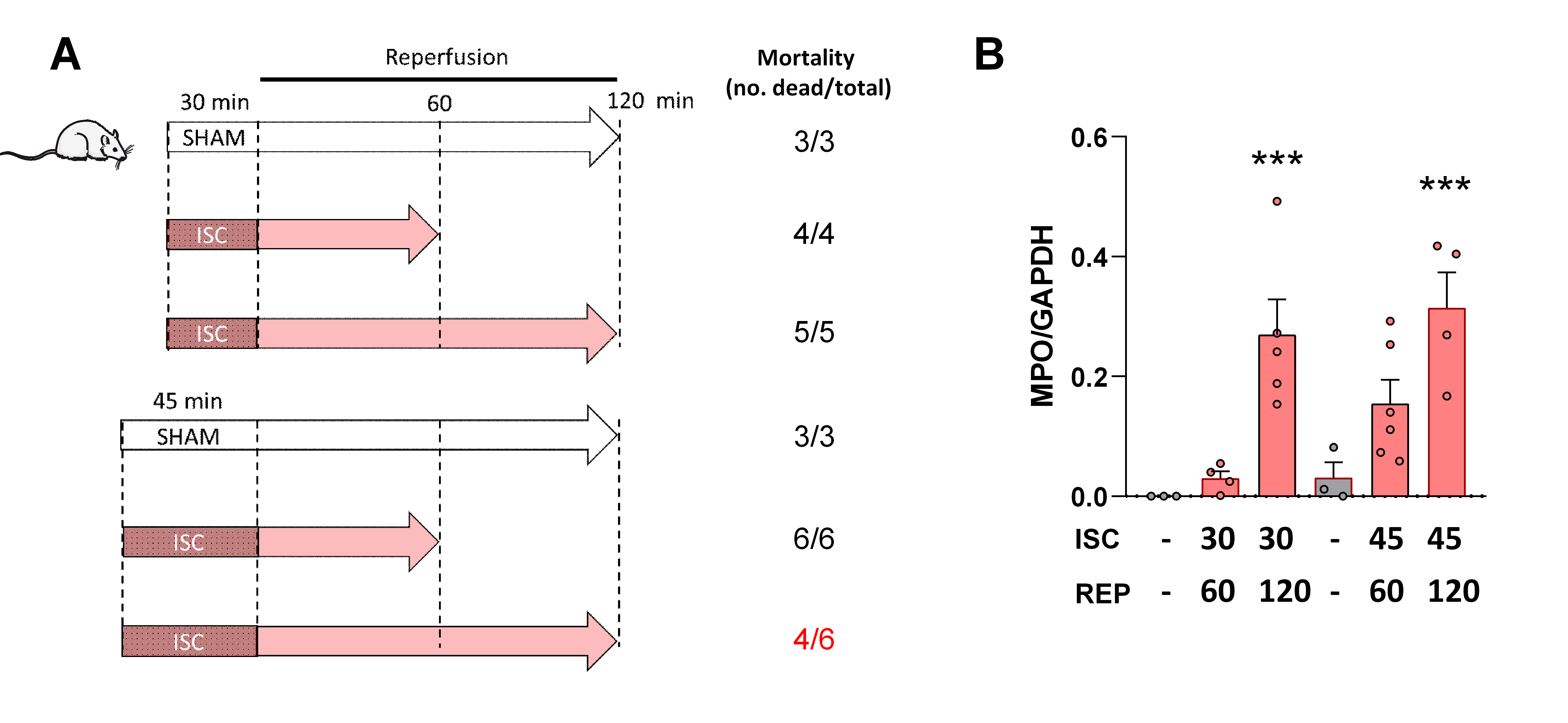

Supplement: Supplementary file 1 [file Image2.TIF]

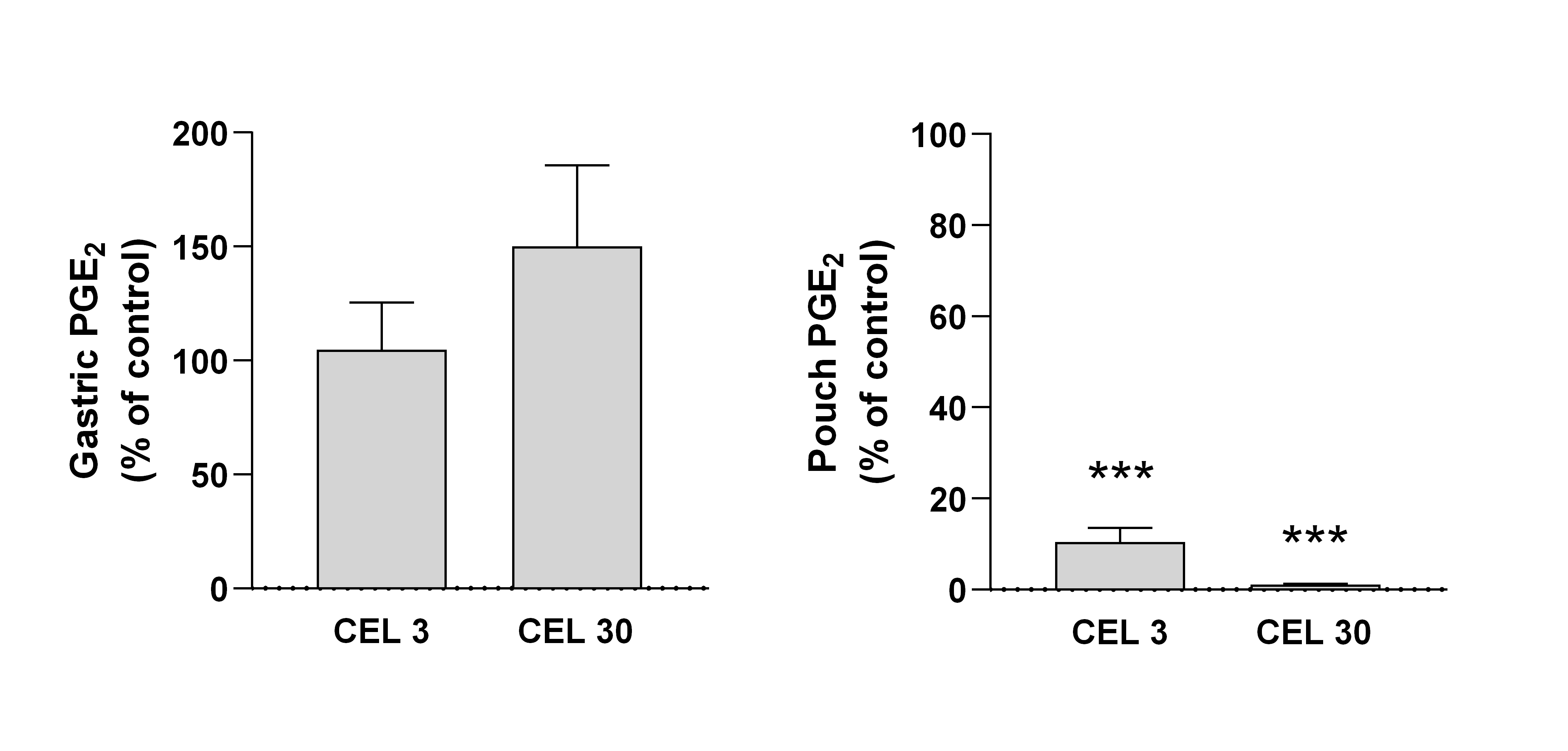

Supplement: Supplementary file 2 [file Image1.TIF]
